# Electricity carbon intensity in European Member States: Impacts on GHG emissions of electric vehicles

**DOI:** 10.1016/j.trd.2017.07.012

**Published:** 2018-10

**Authors:** Alberto Moro, Laura Lonza

**Affiliations:** European Commission, Joint Research Centre (JRC), Via Enrico Fermi 2749, 21027 Ispra (VA), Italy

**Keywords:** Well-to-Wheels (WTW), Life Cycle Assessment (LCA), Battery Electric Vehicle (BEV), Electric Vehicle (EV), Electricity, Greenhouse gas (GHG) emissions

## Abstract

The Well-To-Wheels (WTW) methodology is widely used for policy making in the transportation sector. In this paper updated WTW calculations are provided, relying on 2013 statistic data, for the carbon intensity (CI) of the European electricity mix; detail is provided for electricity consumed in each EU Member State (MS). An interesting aspect presented is the calculation of the GHG content of electricity traded between Countries, affecting the carbon intensity of the electricity consumed at national level. The amount and CI of imported electricity is a key aspect: a Country importing electricity from another Country with a lower CI of electricity will lower, after the trade, its electricity CI, while importing electricity from a Country with a higher CI will raise the CI of the importing Country. In average, the CI of electricity used in EU at low voltage in 2013 was 447 gCO_2_eq/kWh, which is the 17% less compared to 2009. Then, some examples of calculation of GHG emissions from the use of electric vehicles (EVs) compared to internal combustion engine vehicles are provided. The use of EVs instead of gasoline vehicles can save (about 60% of) GHG in all or in most of the EU MSs, depending on the estimated consumption of EVs. Compared with diesel, EVs show average GHG savings of around 50% and not savings at all in some EU MS.

## Highlights

• GHG intensities of electricity consumed in the EU Member States have been calculated for 2013.
• The import of electricity is a critical factor affecting the carbon intensity of a Country.
• CI of electricity consumed at low voltage results, in average, 447 gCO_2_eq/kWh.
• Using EVs, in EU, saves on average 50–60% of GHG emissions compared to internal combustion engines.

## Graphical abstract

Well-to-Wheels emissions of electric vehicles in the Member States of the European Union.

**Figure:**
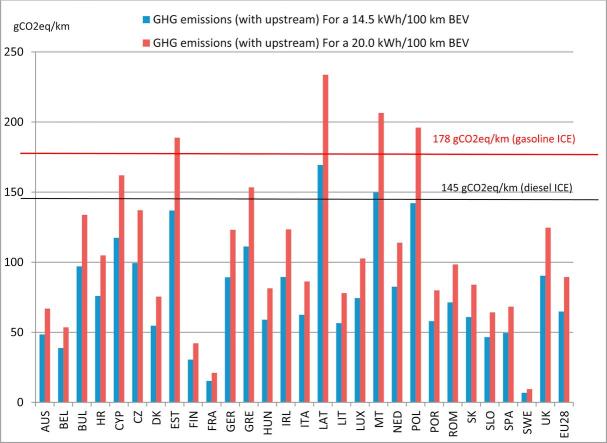

## 1 Introduction

The transportation sector is expected to provide one of the largest contributions to the reduction of greenhouse gas (GHG) emissions according to scenarios supporting policy-making (IEA, 2016a). Environmental policies, to reduce these emissions and diversify energy sources, often promote the use of alternative fuels (including electricity). In order to quantify possible GHG and energy savings, policy makers need to consider sound and shared scientific methodologies allowing comparisons among “cleaner” (with a lower carbon intensity) and conventional technologies. At the regulatory level, Well-To-Wheels (WTW) analysis is the dominant methodology used to assess GHG and energy savings in transport. WTW methodology is used for example by the European Union (EU) for the Fuel Quality Directive (European Union, 2015) and for the Renewable Energy Directive (European Union, 2009), in the United States the Environmental Protection Agency bases its regulatory actions on the WTW approach (EPA, 2007) of the GREET model (Elgowainy et al., 2010). The WTW methodology is used to assess policy options also in other geographic areas, such as in China (Huo et al., 2011).

In the European Union (EU) the WTW analyses performed by the JEC consortium is one of the reference inputs used to provide quantitative information to the regulatory process: the JEC WTW version 4a (JEC, 2014a, JEC, 2014b). This consortium comprises the Joint Research Centre (JRC) of the European Commission, EUCAR, the European council for automotive R&D, and CONCAWE, the research division of the European Petroleum Refiners Association. The most recent JEC WTW report is based, for electricity mix calculations, on 2009 data, and geographically on European average data. The use of the average electricity mix is the most practical way to assess CO2 emissions of EVs (Jochem et al., 2015) so is commonly used for national regulatory purposes, rather than time-dependent approaches (as indicated in Ensslen et al., 2017) more suitable to specific local analyses. This EU averaged approach is correct to address general EU policies but can be considered not satisfactory to optimize policies at national level. Member States (MSs) of European Union are increasingly interested to assess the impact (ex-ante and ex-post) of their own environmental policies. Since inside the European Union there is a strong variability of many techno-economic factors (e.g. the composition of the electric mix), not always EU averaged data allow calculations describing the specificities of single countries. This is particularly true for policies supporting electric vehicles: GHG savings resulting from the use of electric vehicles (EVs) instead of vehicles equipped with internal combustion engines (ICE) can largely vary for different MSs, depending on the carbon intensity of the electricity mix consumed at national or regional level (as said, i.e., by Weiss et al. (2015)).

Performing WTW calculations on the electricity consumed at national level, allowing comparisons between figures characterizing different countries (so using the same methodology), considering also upstream emissions (occurring to extract and transport fuels) and power losses along the grid, is not available in literature; particularly committing is the quantification of the GHG embodied in the electricity traded between Countries.

For example, the International Energy Agency (IEA), provides relevant reports with valuable calculations of GHG content of electricity produced for each Country, even if without upstream emissions (IEA, 2015b); but IEA stops its analysis at the level of gross electricity production. In other words, data refer to production before cross-border trading and not to the electricity actually available for consumption, therefore also not accounting for power losses of the electric grid. This makes these IEA figures not directly useful for assessing GHG savings from the use of EVs instead of ICEs.

Some authors suggested methods to properly consider the GHG content of the electricity traded (Soimakallio and Saikku, 2012), even if implemented with different assumptions. In this paper, the authors present updated (to year 2013) WTW calculations on the CI of EU electricity, by using the same methodology (including upstream emissions and power losses) adopted in the JEC WTW version 4a (2014a). The trade of electricity between countries (defined as in IEA, 2015a) is also considered. The analysis provides details for the carbon intensity of electricity of all EU MSs, from the gross electricity production level to the low voltage level, when electricity is used to recharge electric vehicles, according to the test procedure proposed by UN ECE (2013): “Method of measuring the electric energy consumption of vehicles powered by an electric power train only” (valid also for hybrid electric power trains).

These carbon intensity figures can be used for all the environmental applications requiring WTW input data on electricity. In this paper examples are provided of how carbon intensities affect GHG savings from the use of electric vehicles in EU.

## 2 Material and methods

In this section the JEC WTW methodology (Section 2.1) and the main input data and assumptions adopted for calculations (Section 2.2) are briefly introduced. In Section 2.3 different possible values of carbon intensity are illustrated and discussed. The impact (of CI and quantity) of the electricity imported by a country on the carbon intensity of its electricity supplied is formalized in Section 2.4.

### 2.1 The Well-to-Wheels methodology

The methodology considered in this paper is the Well-To-Wheel (WTW) presented in detail in the JEC WTW report version 4a (JEC, 2014a, JEC, 2014b). This approach allows to quantify the energy required for and the GHG emissions resulting from the production, transport and distribution of conventional and alternative road transportation fuels (Well-To-Tank, WTT), and also to quantify the efficiency of different powertrains (Tank-To-Wheels, TTW). Compared to a comprehensive attributional Life Cycle Assessment (LCA) approach, WTW considers parts of the LCA impact categories “energy consumption” and “GHG emissions”. In the WTW approach, emissions related to the hardware construction, maintenance and decommissioning of fuel producing facilities and vehicles, including materials cycles, are not taken into account, nor are water requirements, acidification or emissions of pollutants if these do not affect the GHG emissions. The GHG taken into account are carbon dioxide, methane and dinitrogen monoxide. The WTW methodology can be seen as a simplified LCA, designed to assess only the energy consumption and the GHG emissions of road transport fuels.

### 2.2 Input data and boundary conditions

The first goal of this article is to detail WTW calculations on the GHG intensity of the electric energy consumed in the European Union, at a Member State level, referring to average values for the year 2013, the most recent and comprehensive statistical set of data available at the time of writing. Main input data and boundary conditions adopted are summarized here.

– Reference year: 2013 (annual average);
– Geographic boundaries: the 28 EU Member States;
– Granularity: Member State level;
– GHG credits assigned for heat produced by CHP (Combined Heat and Power) plants;
– Combustion emission factors from: IPCC (2006);
– Greenhouse gases considered in calculations: carbon dioxide (CO_2_), methane (CH4), Nitrous Oxide (N_2_O);
– Upstream emissions calculated by the JEC with the E3 DB model (LBST, 2017);
– Statistical data for power losses, split between high, medium and low voltage by using ECOINVENT data (Itten et al., 2014).

For each of the EU MSs statistical data were considered for primary energy consumed as input, for electrical energy output produced by power plants and for power losses, relying mainly on the reports of the International Energy Agency (IEA, 2015a, IEA, 2015b, IEA, 2015c, IEA, 2015d). These four reports are complementary and constituting what in this paper is called as “the IEA data set”. This was chosen as main data source because it is consistent with other data sources, such as ENTSO-e and EUROSTAT, 2015, but it provides more details. For aspects connected to electricity trade (Section 2.4) the main IEA data set was integrated with information from ENTSO-e (2014). Input data on primary fuel/energy consumptions has been considered for the same categories adopted in the IEA Electricity Information report (2015a); these are: Hard coal, Brown coal, Peat, Coal gases, Petroleum products, Natural Gas, Solid biofuels, Industrial waste, Municipal waste, Biogases and liquid biofuels, Nuclear energy, Hydropower, Geothermal, Solar, Tide and wave, Wind power. This was applied to both conventional electric and CHP plants.

CHP plants do not use all the primary energy input for electricity production, but part of this energy is used for heating applications; so a GHG credit was considered for the heat produced in output from CHPs according to the “substitution” allocation method adopted both by the IEA and the JEC WTW. The heat produced from the CHPs (IEA statistical data) has been considered as replacing a thermal heating system with average heating efficiencies of 85% for coal, lignite, coke, peat, biomass and waste, and 90% for natural gas, biogas, LPG and oil products, respectively (JEC, 2014a). The CHP credit approach adopted is exactly the same used in the JEC WTW version 4a.

For nuclear power plants the approach in use by main international statistical bodies (IEA, EUROSTAT, IAEA) has been adopted, converting the electric energy produced from nuclear or renewables into an equivalent primary energy value by means of the average thermal efficiency method (e.g. IAEA, 2007). The average thermal efficiency for nuclear power plants adopted is 33%.

The JEC WTW analysis considers GHG emissions occurring in two main “moments” (which are then segmented in further steps), that is: combustion emissions occurring when fuels are burnt and upstream emissions. To calculate the (main) contribution of fuel combustion the IPCC, 2006 Global Warming Potential factors have been used calculated at 100 year horizon (IPCC, 2006), as done by IEA (2015a) for statistical purposes. The upstream emissions are caused by the extraction, refining and transport of the fuels to the power plants and have been calculated using the JEC WTW input data set, relying on the “E3” database (LBST, 2017). The main upstream emission factors adopted are reported in Table 1 and refer to fuel in input to the power plants (gCO_2_eq/MJ). It has to be noticed that these values are placed comfortably among the extremes of other reference studies available in bibliography (Turconi et al., 2013 and ICCT, 2014). For other fuels and renewables such as peat, municipal and industrial wastes, hydropower, geothermal, solar, wind and tidal power the upstream emission factors were considered equal to zero.

**Table 1:** Upstream emission factors adopted.

Fuel	Upstream emission factor
	[gCO_2_eq/MJ]
Hard coal	16.0
Brown coal	1.7
Natural gas	12.8
Petroleum products	10.7
Solid biofuels	0.7
Liquid biofuel	46.8
Biogases	14.9
Nuclear	1.4

Total power losses were accounted on the base of IEA statistical data. However, there are no satisfactory statistical data sources to split losses between the High Voltage (HV), Medium Voltage (MV) and Low Voltage (LV) sections of the electric network. In order to provide results also for the CI of electricity used at HV and at MV (required for the comprehensive quantification of fuel and energy pathways via WTW and LCA calculations), we referred to the broadly adopted ECOINVENT database, relying on the work of Itten et al., 2014. The share of losses adopted does not affect the results for low voltage CI calculations.

### 2.3 Different carbon intensities of electricity

Carbon intensity of electricity can be defined as the GHG emitted for producing or using a certain amount of electricity as shown in equation (1):

(1) CI=GHG emissions/electricity amount

**Fig. 1:**
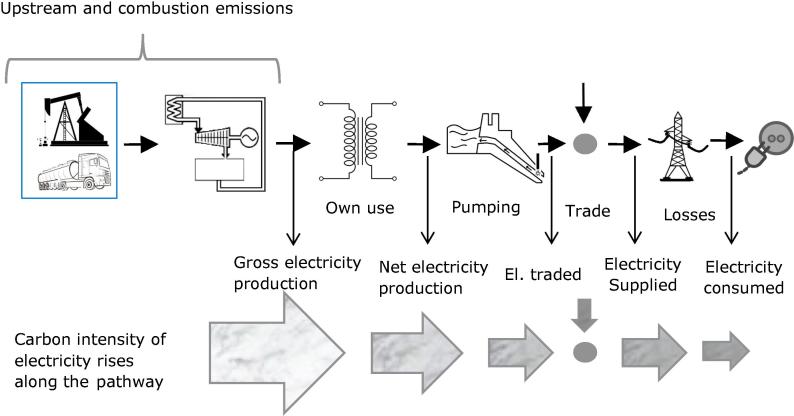
Different Carbon Intensities along the electricity pathway.

Since GHG emissions are expressed in grams (g) of CO_2_ equivalent and the electricity (e.g. produced) is expressed in kWh the consequent carbon intensity (CI) is usually expressed in gCO_2_eq/kWh. However, while comparing different bibliographic sources, it is possible to find different values for (apparently) the same CI of electricity, the same country and the same year. This depends on the stage of the electricity pathway where the CI is calculated: since the amount of GHG emitted is the same, while available electricity along the pathway is decreasing with the losses, carbon intensity rises along the pathway. This is graphically represented in Fig. 1, where larger arrows represent larger amounts of electricity available, and their darker color represent a “dirtier” mix, so a higher carbon intensity of electricity.

In this paper, in order to allow a better comparison with other data sources, we report the carbon intensities for all the following stages of the electricity pathway: gross production, net production, electricity traded, supply post-trade, consumed at high voltage after transmission, consumed at medium voltage after distribution and consumed (by the most of users) at low voltage. For graphical clarity, in Fig. 1 power losses are embedded in a single icon, but our calculations (reported in Table 2) have also this detail.

**Table 2:** Carbon intensities of electricity for EU Member States.

Country	CI of gross electricity production (combustion only)	CI of gross electricity production (with upstream)	CI of net electricity production (with upstream emissions)	CI of electricity traded (with upstream)	CI of electricity supplied (with upstream)	Variation of CI after trade	CI of electricity consumed at HV (with upstream)	CI of electricity consumed at MV (with upstream)	CI of electricity consumed at LV (combustion only)	CI of electricity consumed at LV (with upstream)
	[g/kWh]	[g/kWh]	[g/kWh]	[g/kWh]	[g/kWh]	[%]	[g/kWh]	[g/kWh]	[g/kWh]	[g/kWh]
Austria	133	151	156	170	315	85%	322	325	305	334
Belgium	188	224	233	239	257	8%	261	262	224	267
Bulgaria	507	532	585	601	589	−2%	618	628	636	669
Croatia	231	273	282	285	465	63%	487	494	463	524
Cyprus	646	737	773	773	773	0%	787	792	710	810
Czech Republic	518	545	587	596	640	7%	657	663	643	685
Denmark	316	368	386	386	356	−8%	364	367	328	377
Estonia	1020	1022	1152	1152	840	−27%	878	891	931	944
Finland	171	200	209	209	204	−2%	207	207	181	211
France	66	88	92	93	97	4%	100	101	80	105
Germany	485	534	567	574	588	2%	599	602	558	615
Greece	655	695	755	757	712	−6%	732	739	723	767
Hungary	310	340	368	368	369	0%	383	388	365	407
Ireland	459	533	555	568	570	0%	588	594	530	617
Italy	358	427	444	448	402	−10%	413	417	362	431
Latvia	134	173	185	185	1075	482%	1110	1122	1140	1168
Lithuania	204	246	262	315	358	14%	370	374	331	390
Luxembourg	236	283	283	585	505	−14%	508	509	467	513
Malta	731	831	868	868	910	5%	954	970	908	1032
Netherlands	479	559	582	582	547	−6%	555	558	494	569
Poland	770	847	929	934	911	−3%	937	946	890	980
Portugal	295	346	355	365	357	−2%	372	378	340	400
Romania	356	379	413	416	425	2%	449	457	460	492
Slovakia	173	199	211	215	407	90%	412	414	383	420
Slovenia	315	329	351	361	302	−16%	309	312	291	321
Spain	248	295	305	312	309	−1%	321	325	287	341
Sweden	16	24	25	25	44	74%	45	46	36	47
United Kingdom	469	555	584	591	576	−3%	593	599	526	623
EU 28 average	340	387	407	413	417	1%	428	432	393	447

Note: The measuring unit of the graphic sign [g/kWh] is to be intended as [grams of CO_2_eq/kWh].

As electric vehicles are typically charged at low voltage (UN ECE, 2013), the full sequence of steps from gross electricity production down to electricity actually available for consumption need to be carefully considered. In other words, electricity cross-border trading plays an important role. Results for electricity carbon intensity for each stage listed above and for each EU MSs is reported in Section 3.

### 2.4 Electricity trade and carbon intensity

As shown in Fig. 1, the carbon intensity of the electricity consumed in a country depends also on the CI and amount of electricity traded with other countries. Logically, electricity imported in a country embeds also the GHG necessary for its production, so a WTW (or LCA) calculation aiming at realistically representing the carbon intensity of electricity consumed, should also consider the trade aspect, particularly for countries having high electricity imports. In literature, the aspect of electricity trade is often neglected because of the difficulty in retrieving data, or because the effort required to access and process these data would not fit the scope of analysis. This simplification was done, in example, in the JEC WTW Version 4a (2014a) report, because its focus is on EU average values and the difference between electric energy supplied before and after extra-EU imports was, in 2009, less than 0.5% (15 TWh over a total of 3046 TWh: the EU electric grid is quite self-sustaining). This approximation is still valid, for the EU as a whole, and from Table 2 it is possible to appreciate that the difference, in 2013, between CI of electricity traded (413 gCO_2_eq/kWh) and supplied (417 gCO_2_eq/kWh) is only of 1%

In order to provide details on the carbon intensity of electricity for a specific country (the EU MSs, in our case) considering cross-border trading of electricity becomes necessary. The approach we adopted can be explained as follows.

First, we need to clarify at which point in the electricity pathway trade occurs: according to our main reference source from IEA, this happens after the pumping step. So the electricity (el.) supplied to a national network, considering the trade, is defined by the IEA with the equation (2):

(2) el.supplied=el.net production-pumping+Imports-Exports

For all these terms presented in equation (2) we used IEA statistical data.

In order to calculate the CI of the electricity supplied (post trade) in a country it is possible to use equation (1), considering in the denominator the result of equation (2), and in the numerator the value of total GHG emissions embedded in the electricity supplied, calculated according to equation (3):

(3) Total GHG=Combustion GHG+Upstream GHG-Exported GHG+Imported GHG

Combustion and upstream GHG emissions are the same values used to calculate the CI of electricity produced in each country, the exported GHG is simply the product between the CI of electricity traded (the values we used are reported in Table 2) multiplied by the amount of electricity exported, while the Imported GHG can be calculated by Eq. (4).

(4) $$\overset{N}{\underset{i=1}{\sum }}\mathrm{el}.\mathrm{Import}\;\mathrm{from}\;\mathrm{Country}\;\text{"“"}\text{i}\text{"”"}\;*\;\text{CI el}.\text{traded Country}\;\text{"“"}i\text{"”"}\;$$

where for each country it is necessary to consider the sum-product of all the amount of electricity traded (el. Import from Country “i”) and the respective Carbon Intensities (CI el. traded Country “i”). We performed these calculations for each EU MSs, considering all the Import-Exports from and towards all the involved EU and extra-EU countries. Carbon intensities of extra EU countries are calculated at a lower level of detail compared to EU Countries described in Section 2.2 (so they are not reported in Table 2) but using Country specific combustion emissions from IEA, 2015b with average EU upstream emissions (13.9% of the combustion emissions). This lead to the following CI of electricity traded (with upstream emissions): Norway: 9 gCO_2_eq/kWh; Switzerland: 29 gCO_2_eq/kWh; Turkey: 528 gCO_2_eq/kWh; Belarus: 513 gCO_2_eq/kWh; Russian federation: 517 gCO_2_eq/kWh; Serbia: 939 gCO_2_eq/kWh; Ukraine: 586 gCO_2_eq/kWh; “Non-specified” and “Others”: 690 gCO_2_eq/kWh.

Quantitative results of these calculations are reported in Section 3. These, considering the approximations discussed in Section 4, rely on an integrated set of statistical data elaborated from IEA, 2015a, IEA, 2015b, IEA, 2015c, IEA, 2015d and ENTSO-e (2014).

## 3 Results

Table 2 reports the results of our calculations for the different values of carbon intensity described in Section 2.3, calculated for each EU MSs for the year 2013. Calculations have been performed according to input data and boundary conditions detailed in the Sections 2.2, 2.4.

The second and third columns of Table 2 show the Carbon Intensity (CI) for one kWh of gross electric energy produced; data are provided, to allow comparison with other sources, in two modes: with and without upstream emissions. As a “sanity check”, we compared our figures for the CI of gross electricity production “with combustion emissions only” with the IEA (2015b) data set, finding an average difference of only 1%.

It is interesting to note that there is a difference between the CI of electricity traded and supplied in several Member States (see column: “Variation of CI after trade” in Table 2). After the trade some Countries are worsening (raising) their electric CI while others are improving it (see discussion in Section 4.1).

For power losses occurring in the electric grid we considered, as total value, the statistics from IEA, 2015a, IEA, 2015d, split between the HV, MV and LV sections according to the ECOINVENT approach (relying on: Itten et al., 2014).

Also for the CI of electricity consumed at low voltage, we provided two values: one with and one without upstream emissions. The value considering combustion emissions only is fully consistent with IEA data set.

## 4 Discussion and application on electric vehicle emissions

Carbon intensity of the electricity mix is widely used in LCA and WTW applications with electricity being a key input in numerous industrial processes. The WTW can be considered as a simplified methodology, compared to the LCA, but, for the carbon intensity of electricity, results are very similar. In a previous study, Moro and Helmers (2015) assessed and demonstrated the alignment of the JEC WTT data set for electricity and the main LCA data sets in the EU and for the year 2009.

The upstream emissions we used for fossil fuels (for extraction, transport and refining of the fuels) are the same adopted in the JEC WTT Version 4a (2014a) report. For oil and natural gas other studies provide different upstream values (ICCT, 2014, COWI, 2015), so we performed a sensitivity analysis by replacing, in our calculations, the WTW upstream emissions with those from ICCT and COWI. Both studies rely on a model (OPGEE) developed by Stanford University (El-Houjeiri, Brandt and Duffy, 2013) in the USA, and recently used to compute upstream emissions for the crude slate relevant for the EU. Crude oil carbon intensity is, according to these studies, 10.0 gCO_2_eq/MJcrude (5.1 gCO_2_eq/MJcrude in JEC WTT Version 4a). The sensitivity analysis we performed on the CI of electricity consumed in EU at LV show crude oil upstream emissions differing, for new OPGEE and JEC WTT Version 4a data sets, of less than 0.2%. The same sensitivity analysis between OPGEE and the JEC WTT-Version 4a for Natural Gas upstream emissions leads to a difference in the order of 0.8%.

Important bibliographic sources (e.g. IEA, 2015b) do not consider upstream emissions but only combustion emissions and limit analysis at the level of gross electricity production. Since we provided figures (in Table 2) also for carbon intensities referring to combustion emissions only and we verified our values are fully in line (differing on average of 1%) with IEA (2015b), it is possible to use our results for CI of electricity at LV, in conjunction with IEA data sets.

In order to assess the evolution in time of the EU electric mix, a comparison was performed also between the CI of our 2013 EU-28 average mix (447 gCO_2_eq/kWh at LV) and 2009 EU27 JEC WTT values (540 gCO_2_eq/kWh), verifying that the European Union has reduced, on average, the carbon intensity of its electric mix by 17%.

While calculating the impact of cross-border trading on GHG intensities of electricity supplied for all the EU MSs, the challenge is on finding consistent data sets for all the MSs; particularly, for non-OECD Countries, which are unfortunately less well or not homogeneously described by IEA statistics.

An approximation in calculations involving electricity trade is introduced by the fact that statistical data only describe electricity trade between neighboring countries, and data on electricity trade can have “some distortion” (IEA, 2015a, p. II.5). A further approximation is the fact of considering, as CI of the imported electricity, the CI of the average annual production of the exporting countries. This, probably, does not correctly represent the mix of the electricity volumes which are actually traded. Typically, this is a surplus of production for specific energy sources: probably baseload power plants (e.g. nuclear) during the night or renewables in excess during the day. However, we consider this approach as the most accurate and feasible based on the statistical data publicly available.

### 4.1 Considerations on the impact of electricity trade in the national CI

This section discusses some general aspects of possible effects of the electricity cross-border trading on net national CIs based on the results presented in Table 2 columns: “CI of electricity traded (with upstream)”, “CI of electricity supplied (with upstream)” and “Variation of CI after trade”.

It is important to note that the part of the trade affecting the CI of a country is only the electricity imported (Imports). From equation (1) it is possible to understand why Exports do not affect at all the carbon intensity of electricity consumed.

**Fig. 2:**
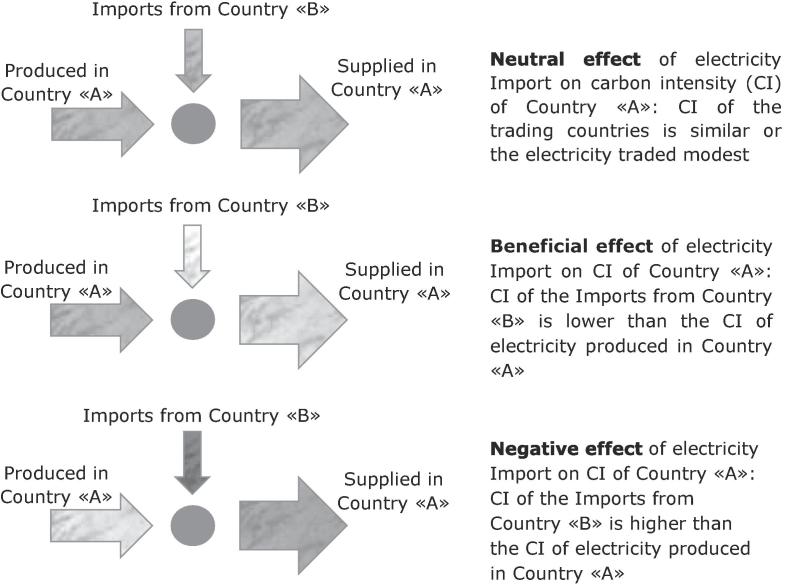
Visual representation of the impact of Imports on carbon intensity of electricity supply.

In general, it is possible to identify three typical cases of electricity cross-border trading affecting the carbon intensity of the electricity supplied in a given Country, which we call “A”, importing energy from another Country, named “B” (as in Fig. 2).

The first case is a “neutral effect” of electricity Imports; this happens when the CI of the electricity imported from Country B is similar to that of the electricity produced in Country A, or when the amount of electric energy imported is modest. This happens in the majority of the EU MS and for the EU as a whole, where Imports change the CI of electricity supplied by approximately 1% only.

The second typical case is a “beneficial effect” of trade; it happens when Country A produces electricity with a CI higher (arrow with a darker color in Fig. 2) than the electricity imported from Country B (lighter color in Fig. 2), so the environmental effect of the Import is, for Country A, to “clean” its own electric mix. This effect is evident, for example, for Estonia, producing electricity mainly from a very GHG intensive source (peat) and importing it from Finland, which relies on renewables and nuclear energy.

The third typical case is the “negative effect” of trade; it happens when Country A with a “cleaner” electric mix imports from Countries with higher CIs (or “dirtier” mix). This is the case of Latvia, worsening its mix because of relevant imports from Estonia, but also of Sweden, which has such a “clean” electricity production that whatever Import, also from the relatively “clean” Norway and Denmark, can just worsen its CI.

### 4.2 Assessment of GHG savings from the use of battery electric vehicles

As an example of possible application of the figures presented in Section 3, this section recalls some useful concepts and presents some examples of GHG emissions or GHG savings calculations attributable to the use of electric vehicles by using the Carbon Intensities of Table 2. Please note that EVs considered are battery electric vehicles (BEV).

GHG savings from the use of BEVs can be seen as the difference between GHG emissions from the considered BEVs and GHG emissions due to the use of the internal combustion engine (ICE) vehicle alternative (both expressed in gCO_2_eq/km). These savings are represented by equation (5):

(5) GHG savings=GHG from the use of ICE-GHG from the use of BEV

The GHG emissions from the use of ICEs are not varying across EU; while the GHG emissions profiles of BEVs depend on the (national) CI of electricity used. By means of the CIs reported in Table 2, it is possible to assess the GHG emissions per km from the considered battery electric vehicle according to equation (6):

(6) BEV emissions=CI electricity∗BEV consumption/100

where

BEV emissions are the GHG emissions per km of a BEV, expressed in [gCO_2_eq/km].
CI electricity is the carbon intensity of the electric energy used at LV to recharge the BEV, expressed in [gCO_2_eq/kWh].
BEV consumption is the BEV electric energy consumption (from literature or data sheets), expressed in [kWh/100 km].

The JEC TTW Version 4a study (JEC, 2014b) considers as reference vehicle, an averaged or “virtual” car representing the most widespread European C—segment 5-seater European sedan available in the year 2010. For gasoline engines, the reference vehicle is equipped with a 1.4-liter direct injection spark ignition (DISI) ICE, resulting in emissions of 178 gCO_2_eq/km; for diesel engines the reference vehicle is equipped with a 1.6-liter direct injection compression ignition (DICI) ICE emitting 145 gCO_2_/km. These data, well shared among the scientific community, are referring to neat fuels (not blended with biofuels) and provided at EU average level. However, little changes would be experienced at a EU Member State level because most of the emissions, for gasoline and diesel, is due to their combustion, and upstream emissions are mainly driven by factors not varying with the specific EU Member State as detailed in JEC, 2014c (e.g. for gasoline total emissions are: 87.1 gCO_2_eq/MJ of which: combustion 73.4 gCO_2_eq/MJ; crude oil production and transport to the EU: 5.6 gCO_2_eq/MJ; refining: 7.0 gCO_2_eq/MJ; distribution and dispensing: 1.2 gCO_2_eq/MJ).

All the JEC TTW fuel consumption figures are evaluated on the basis of the current European type-approval cycle NEDC (New European Driving Cycle). The BEV consumption is measured according to the UN ECE 101 (2005) Regulation, so it also includes the charging losses occurring during the “slow” charge procedure (called “normal overnight charge” in the UN ECE 101), by using the on board charger of the BEV.

Possibly due to the novelty of EVs in general and to a small market size, the TTW data describing energy consumption of EVs are not as homogeneous as their ICEs counterparts. According to the JEC TTW Version 4a study, the average BEV has an electricity consumption of 14.5 kWh/100 km, while other sources provide different values. Some authors adopt as standard consumption the data sheet of some specific vehicle (18.7 kWh/100 km is used by Ensslen et al. (2017) while VTT (2013) adopts the 21 kWh/100 km for the Nissan Leaf), others (e.g. ECOINVENT) model average real-world consumptions (19.9 kWh/100 km according to Del Duce, Gauch and Althaus, 2016), other authors measure, in laboratory, consumptions occurring for a specific vehicle under different driving cycles and operating conditions (15.7 kWh/100 km for a NEDC cycle at 25 °C according to De Gennaro et al., 2015), while others consider the composition of the actual EV fleet. This last is the case of IEA models (IEA, 2016b) considering 2015 consumptions of EVs varying, in some EU MSs, between 17.1 kWh/100 km and 21.5 kWh/100 km (weighted average: 20.0 kWh/100 km). Since the scope of this section is just to provide examples, we performed calculations for two relevant kinds of BEVs: a (JEC) BEV consuming 14.5 kWh/100 km and a (IEA) BEV consuming 20.0 kWh/100 km. For specific applications, the reader can use different values representing ICE or BEV consumptions.

**Fig. 3:**
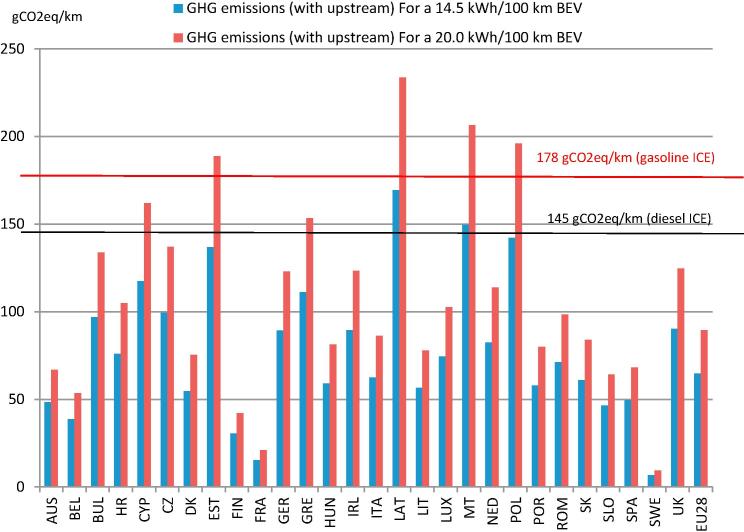
GHG emissions due to the use of electric vehicles in the European Union.

According to equation (6), based on the CI of electricity used at LV in the EU MSs (Table 2) and for the two selected BEVs (in blue the BEV consuming 14.5 kWh/100 km and in red the BEV consuming 20.0 kWh/100 km) results of GHG emissions calculations are visualized in Fig. 3.

Fig. 3 shows how GHG emissions from the use of EVs varies across the EU: while in Sweden the use of BEV would produce only 7–9 gCO_2_eq/km, in Latvia EVs emit 169–234 gCO_2_eq/km and the EU average is 65–89 gCO2eq/km (the first number of these intervals refer to the 14.5 kWh/100 km BEV while the second to the 20.0 kWh/100 km BEV). According to these figures, the use of BEV in countries relying on big shares of nuclear or renewable electricity would contribute to reducing GHG emissions at the national level, while, in countries with a highly carbon-intense electricity mix, electric cars would not necessarily contribute to GHG emission reduction targets than relying on ICE vehicle fleets.

A visual comparison of GHG savings from the use of BEVs instead of conventional ICE vehicles in different MSs can be done by assessing the distance between the vertical bars representing GHG emissions from BEV and the horizontal lines representing the reference emissions from ICEs.

By comparing the GHG emissions from the use of the BEV consuming 14.5 kWh/100 km with gasoline-fuelled ICEs (178 gCO_2_eq/km), GHG savings are achieved in all the MSs (64% of emissions are saved, on EU average); compared to diesel-fuelled ICEs (145 gCO_2_/km) this BEV does not always achieve GHG savings, although the EU average is 55%.

The second BEV analyzed, consuming 20.0 kWh/100 km, is not GHG saving in some MSs also in comparison with a gasoline-fuelled ICE (50% of GHG savings on average), while in comparison with a diesel-fuelled ICE this BEV is not producing GHG savings in six MSs, with an average of savings falling to 38%.

It is possible to summarize these results by stating that, based on TTW data from JEC (2014b) and IEA (2016b) for BEVs, in the EU and in 2013, savings were on average about 60% of GHG compared to gasoline and 50% compared to diesel ICEs respectively.

For completeness purposes, we compared ICE vehicles using blends of biofuels (instead of neat fossil fuels) verifying that, at the blending levels available on the EU market (5% v/v of ethanol blended in fossil gasoline, and 7% v/v of biodiesel blended in fossil diesel fuel), this does not significantly affect the GHG savings of BEVs.

The calculations performed in this paper rely on the JEC WTW methodology. In order to (roughly) compare these WTW data with LCA results we refer to our previous work (Moro and Helmers, 2015). There, we demonstrated that the main difference between GHG emissions from EVs calculated with the WTW and the LCA methodologies relies essentially in the battery pack. Considering also the LCA emissions to produce and dispose the batteries used during the life of the EV, WTW figures on GHG savings would decrease by about 10%.

## 5 Conclusions

In order to reduce transportation emissions and diversify energy sources, alternative fuels are being promoted at the global, EU and national level. Legislation often relies on values and calculations based on the WTW methodology. In order to assess GHG emissions from electric vehicles and GHG savings in comparison with ICE vehicles it is necessary to know the carbon intensity of electricity consumed to recharge BEVs.

In this paper, we provide figures on the carbon intensity of electricity consumed in European Union in 2013 based on the most recent full set of statistical data available. Details are provided for the CI of electricity available for consumption in each EU Member State, hoping to provide useful figures and insights to national and local policy makers, helping them to better assess GHG savings from the use of EVs in their country and to better tailor their alternative fuels policies.

The effect of the electricity cross-border trading on the CI of electricity supplied and available for consumption in the various Member States has been calculated and analyzed: after the electricity trade there are countries worsening (raising) their CI and other countries improving it (lower CI). This depends on the CI and on the amount of the imported electricity: imports from a country with a lower CI of the electricity mix will result in a lower CI for the importing country; conversely, importing from a country with a higher CI will raise also the CI of electricity supplied in the importing Country.

The carbon intensity calculations were performed in two main modes: by considering GHG combustion emissions only and by considering combustion and upstream emissions; results were provided for both cases thus allowing different uses of our results and analysis. Results calculated for combustion emissions only are fully in line with the IEA input data and the IEA CO_2_ emission calculations. These results are therefore available to be used as a IEA-consistent data set. Conversely, results including upstream emissions are consistent with the JEC WTW methodology used as one of the reference inputs to the EU regulatory framework. Compared to 2009 JEC WTW data sets, 2013 carbon intensity of the EU electricity mix is 18% lower, showing a consistent decarbonisation trend of the electricity mix in Europe.

Lastly, we provided some examples of the use of these CIs to assess GHG savings when using BEVs. For the pair of reference data sets used, we estimated that BEVs are saving GHG in all or in the most of EU MS compared to gasoline-fuelled ICEs. Compared to diesel-fuelled ICEs, this evidence is not as thorough. In general, it is possible to say that, in the European Union and in 2013, BEVs allowed potential GHG savings compared to ICEs in the range of 50–60%.

Assuming a continuing decarbonisation trend of the EU electricity mix, BEVs are expected to have lower GHG emissions profiles than any ICEs in the near future.

## Glossary

BEV: Battery Electric Vehicle
CI: Carbon Intensity
CHP: Combined Heat and Power plants
ENTSO-e: European Network of Transmission System Operators for Electricity
EU: European Union
EU-27: European Union with 27 Member States
EU-28: European Union with 28 Member States (including Croatia)
EV: Electric Vehicle
GHG: Greenhouse Gas
GREET: Greenhouse gases, Regulated Emissions, and Energy use in Transportation
gCO_2_eq/km: Grams of CO_2_ equivalent per kilometer
gCO_2_eq/kWh: Grams of CO_2_ equivalent per kiloWatthour
ICE: Internal Combustion Engine
IEA: International Energy Agency
JEC: JRC-Eucar-Concawe consortium
JRC: Joint research Centre of the European Commission
kWh: kilowatt-hour
LCA: Life Cycle Assessment
LV: Low Voltage
MS: Member State of the European Union
TTW: Tank to Wheel
WTW: Well to Wheel
WTT: Well to Tank
